# Supplementary material for: Opioid- and NMDA-receptor-dependent neural plasticity mediates long-term analgesia from motor cortical stimulation
Source: bioRxiv. 2026 Jul 7:2026.07.01.735554. Preprint. [Version 1] doi: 10.64898/2026.07.01.735554 (PMC13370358; doi:10.64898/2026.07.01.735554)
Supplement: Supplement 1 [file NIHPP2026.07.01.735554v1-supplement-1.pdf]

bioRxiv preprint doi: https://doi.org/10.64898/2026.07.01.735554; this version posted July 7, 2026. The copyright holder for this preprint (which was not certified by peer review) is the author/funder, who has granted bioRxiv a license to display the preprint in perpetuity. It is made available under a CC-BY 4.0 International license.

Supplemental Figure 1

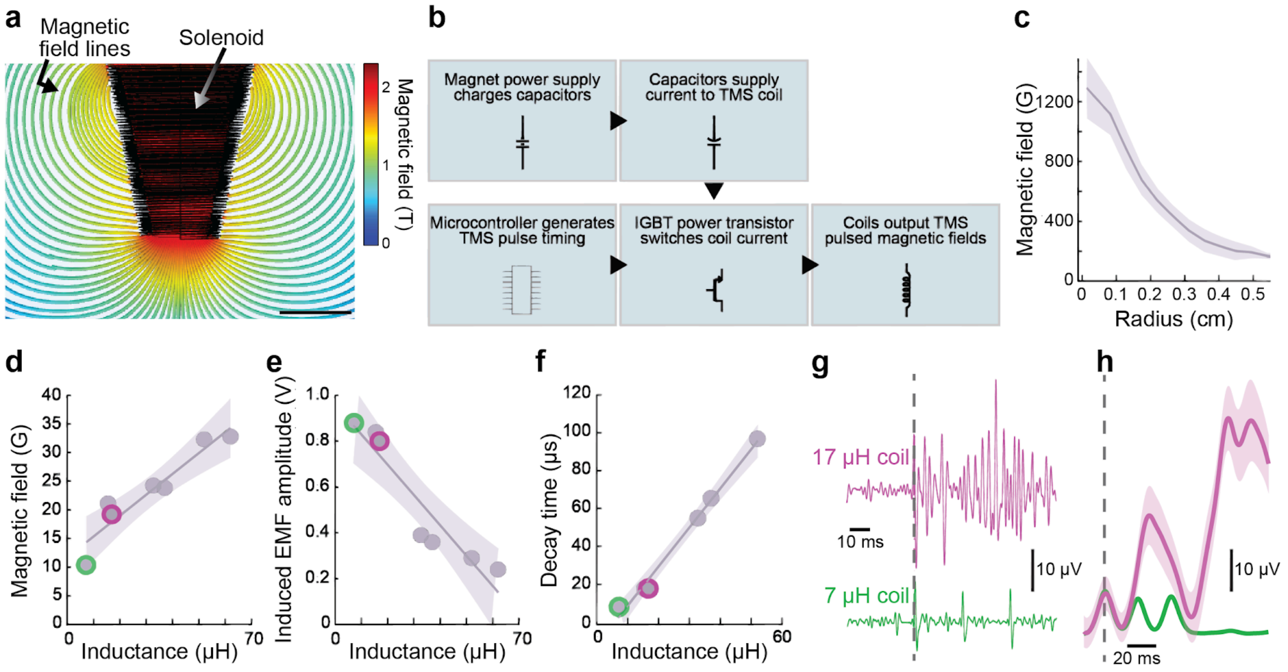

Supplemental Figure 1 | Design and testing of miniaturized TMS coils.

(a) Map of the magnetic field lines (colored lines) generated by a miniTMS coil with three layers of 0.4-mm-diameter Cu wire wound around a powder-iron core (relative permeability of 75, driven at 100 A), as computed with the COMSOL finite-element physics simulation package. Colors indicate magnetic field amplitudes. Scale bar: 2 mm.

(b) Basic schematic of the driver electronics for the miniTMS apparatus. A power supply (100 V; 2 A) charges a capacitor bank while a microcontroller generates precisely timed trigger pulses. The supply current from the capacitor bank and the trigger pulses converge on an insulated-gate bipolar transistor (IGBT) switch that delivers monophasic, square current pulses to the coil, creating the rapidly changing magnetic field values needed to depolarize neurons. Compared to traditional TMS coils that have an air-core, our coils substantially reduce the challenges of heat production, owing to the concentration of the magnetic field in the iron core and the use of brief (~50 μs) pulses (**Fig. 1b**).

bioRxiv preprint doi: https://doi.org/10.64898/2026.07.01.735554; this version posted July 7, 2026. The copyright holder for this preprint (which was not certified by peer review) is the author/funder, who has granted bioRxiv a license to display the preprint in perpetuity. It is made available under a CC-BY 4.0 International license.

(c) Mean amplitude of the magnetic field from a miniTMS coil, plotted as a function of the radial distance from the coil axis, as measured with a Hall probe positioned axially ~1.0 mm beneath the core tip. Shading: s.e.m. across sampled grid points within each radial bin (n=4–21 points per bin).

(d–f) Electromagnetic properties of miniTMS coils with inductances between 7–62 μH. Plotted are the **(d)** magnetic field strength (measured with a Hall probe axially displaced ~1.0 mm from the tip of each coil), **(e)** induced electromotive force (EMF, measured with a 2-mm-diameter current loop axially displaced ~1.0 mm from the coil tip), and **(f)** EMF decay time (measured from coil current decay, recorded with a 0.1 Ω current-sensing resistor in series with the coil), shown as functions of coil inductance (measured with an electronic inductance meter). To vary the coil inductance value, we changed the number of wire wraps (2 *vs.* 3) and the winding geometry around the iron core. Data points encircled in green or pink indicate the two coils for which EMG traces are shown in **g**. Solid lines: Linear regression fits (Shading: ± 95% C.I.).

(g, h) Example raw EMG traces (bandpass-filtered between 10 Hz–20 kHz by the EMG amplifier), **g**, and trial-averaged traces of the EMG envelope (n=45 trials; bandpass filtered (100–500 Hz), rectified, and then low-pass filtered (50 Hz)), **h**, as recorded in the vibrissa protractor muscle. Traces are plotted as functions of time relative to delivery of the TMS pulse (dashed vertical line) to the contralateral motor cortex (**Methods**). TMS pulses delivered in triplets (20-ms-interpulse interval) were delivered at pseudorandomly chosen times with inter-pulse-intervals of 30–50 ms. We used a miniTMS coil with either a 17 μH (magenta traces) or a 7 μH (green traces) inductance. Based on the greater EMG signals elicited by the 17-μH-coil, we used coils of this design and inductance value for all subsequent experiments described in this paper. Shading in **h**: s.e.m. over 45 trials. (Note that s.e.m. values are too small to see on the lower trace).

bioRxiv preprint doi: https://doi.org/10.64898/2026.07.01.735554; this version posted July 7, 2026. The copyright holder for this preprint (which was not certified by peer review) is the author/funder, who has granted bioRxiv a license to display the preprint in perpetuity. It is made available under a CC-BY 4.0 International license.

Supplemental Figure 2

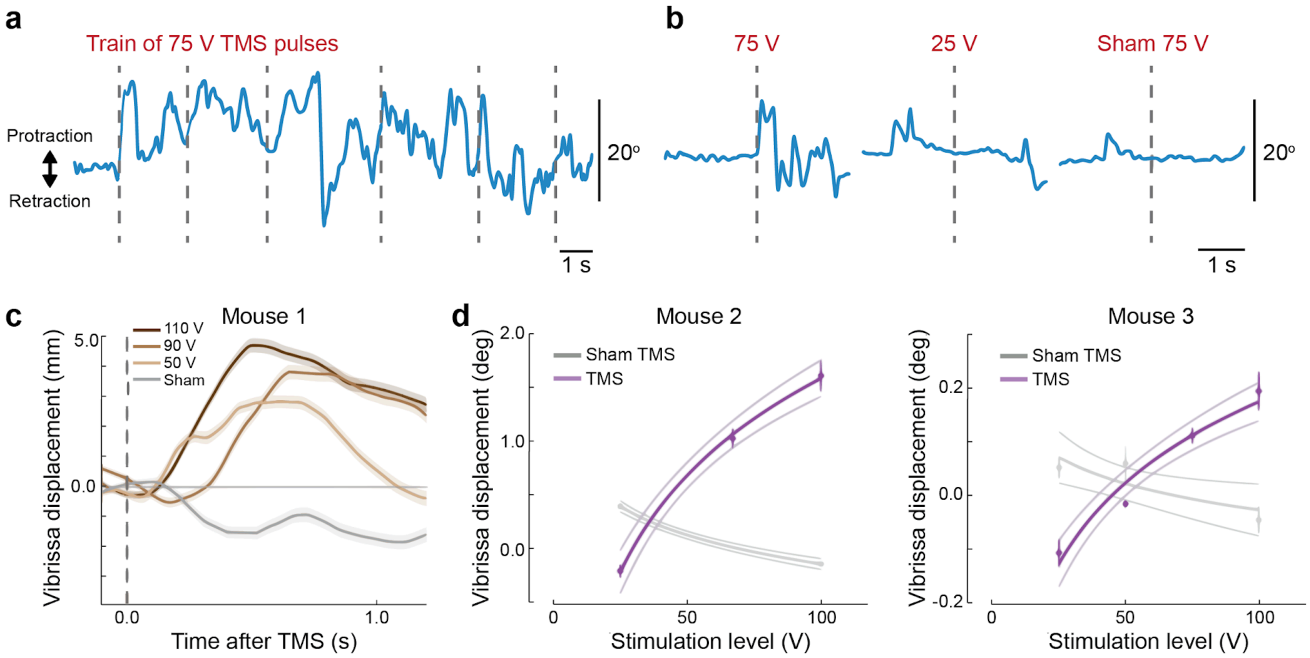

Supplemental Figure 2 | Vibrissa movement increases with the intensity of TMS.

(a, b) Traces of vibrissa angle relative to the mouse's head, as determined by machine vision analyses of behavioral videos taken during stimulation of the contralateral motor cortex with a 17-µH miniTMS coil. **(a)** Example trace of vibrissa angle during a series of TMS pulses delivered with a drive voltage of 75 V. **(b)** Traces showing vibrissa angle in response to TMS pulses with drive voltages of either 75 V or 25 V. In the sham (75 V) stimulation condition, the miniTMS coil was held ~5 cm above the mouse's head. Dashed vertical lines mark times at which TMS pulses were applied to the motor cortex.

(c) Displacements of the vibrissa base for an example mouse (mean ± s.e.m.; n=50 trials per stimulation intensity) evoked by single TMS pulses delivered with drive voltages of 110 V (dark brown), 90 V (light brown), or 50 V (peach) TMS, or by sham stimulation with a 50 V drive voltage (gray, TMS coil positioned ~5 cm above the head to maintain the auditory stimulus associated with a TMS pulse while precluding effective magnetic stimulation of the brain).

bioRxiv preprint doi: https://doi.org/10.64898/2026.07.01.735554; this version posted July 7, 2026. The copyright holder for this preprint (which was not certified by peer review) is the author/funder, who has granted bioRxiv a license to display the preprint in perpetuity. It is made available under a CC-BY 4.0 International license.

(**d**) Vibrissa displacements evoked by a single pulse of real or sham TMS, plotted as a function of peak stimulation voltage (mean ± s.e.m.; n=50 trials per datum). The graph format, parametric fits, and experimental details are the same as those in **Fig. 1d**.

bioRxiv preprint doi: https://doi.org/10.64898/2026.07.01.735554; this version posted July 7, 2026. The copyright holder for this preprint (which was not certified by peer review) is the author/funder, who has granted bioRxiv a license to display the preprint in perpetuity. It is made available under a CC-BY 4.0 International license.

Supplemental Figure 3

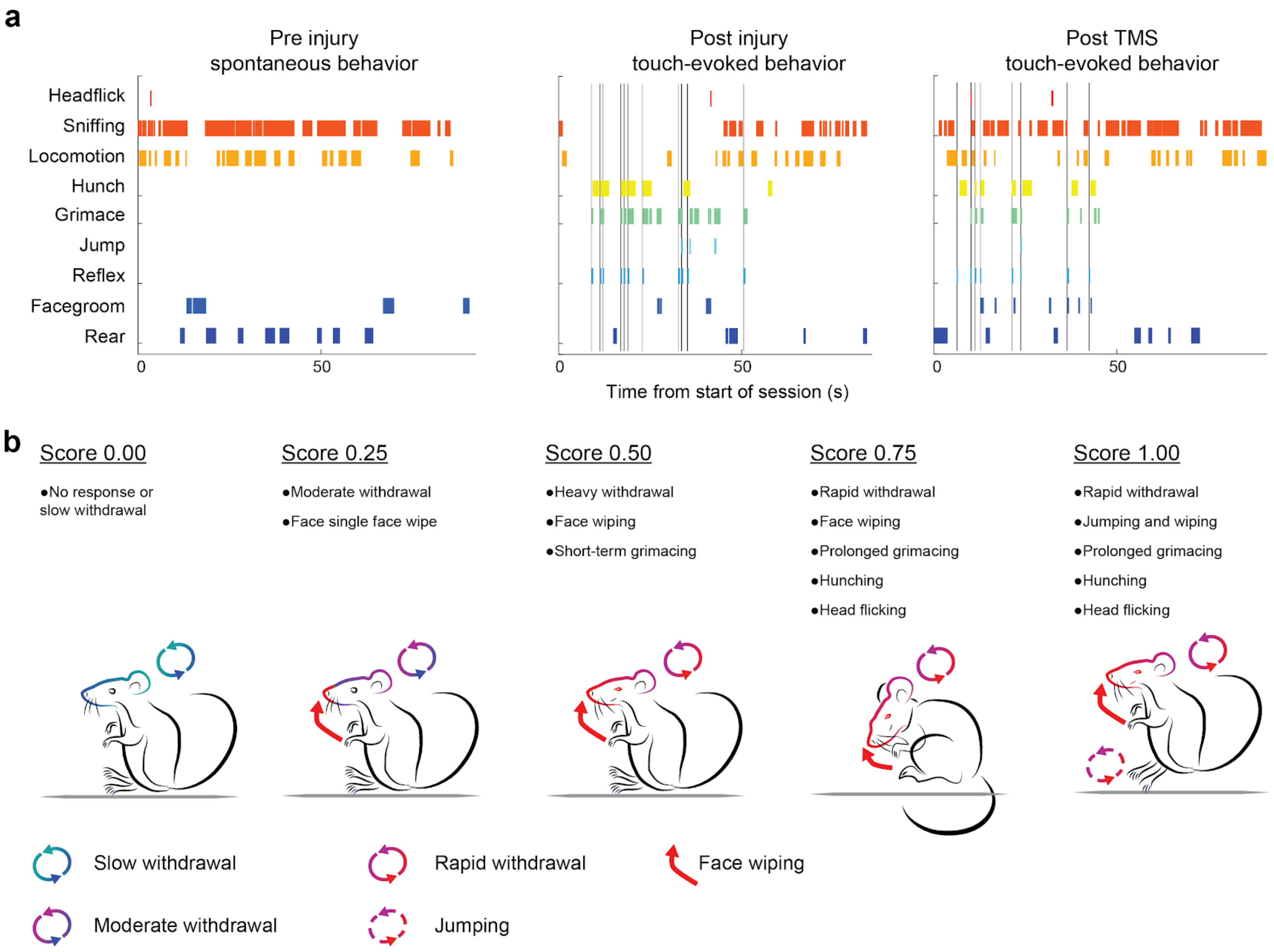

Supplemental Figure 3 | Numeric grading of mouse nocifensive behaviors in response to mechanical stimuli applied to the face.

(a) Example raster plots showing results obtained through visual scoring of mouse behavior, revealing both pain-related (head flick, hunching, grimace, jump, reflex, and facial grooming) and healthy behaviors (sniffing, locomotion, and rearing). Each plot shows data from the same individual mouse engaged in spontaneous behavior during an initial, pre-injury testing session (*left*), during sensory testing after the facial nerve injury (*middle*), and 2 days after TMS treatment applied at 75% of the

bioRxiv preprint doi: https://doi.org/10.64898/2026.07.01.735554; this version posted July 7, 2026. The copyright holder for this preprint (which was not certified by peer review) is the author/funder, who has granted bioRxiv a license to display the preprint in perpetuity. It is made available under a CC-BY 4.0 International license.

motor threshold (*right*). Vertical lines in the middle and right panels mark times at which the von Frey filament was applied to the mouse's face.

(b) Schematic illustration of the scoring method used to grade nocifensive responses and orofacial pain. Each touch-evoked response was assigned a score between 0 and 1 based on the number, duration, and type of nocifensive behaviors. Colored ellipses indicate the relative velocity of withdrawal or jumping. Red arrows and facial expressions depict face wiping and grimacing, respectively.

bioRxiv preprint doi: https://doi.org/10.64898/2026.07.01.735554; this version posted July 7, 2026. The copyright holder for this preprint (which was not certified by peer review) is the author/funder, who has granted bioRxiv a license to display the preprint in perpetuity. It is made available under a CC-BY 4.0 International license.

Supplemental Figure 4

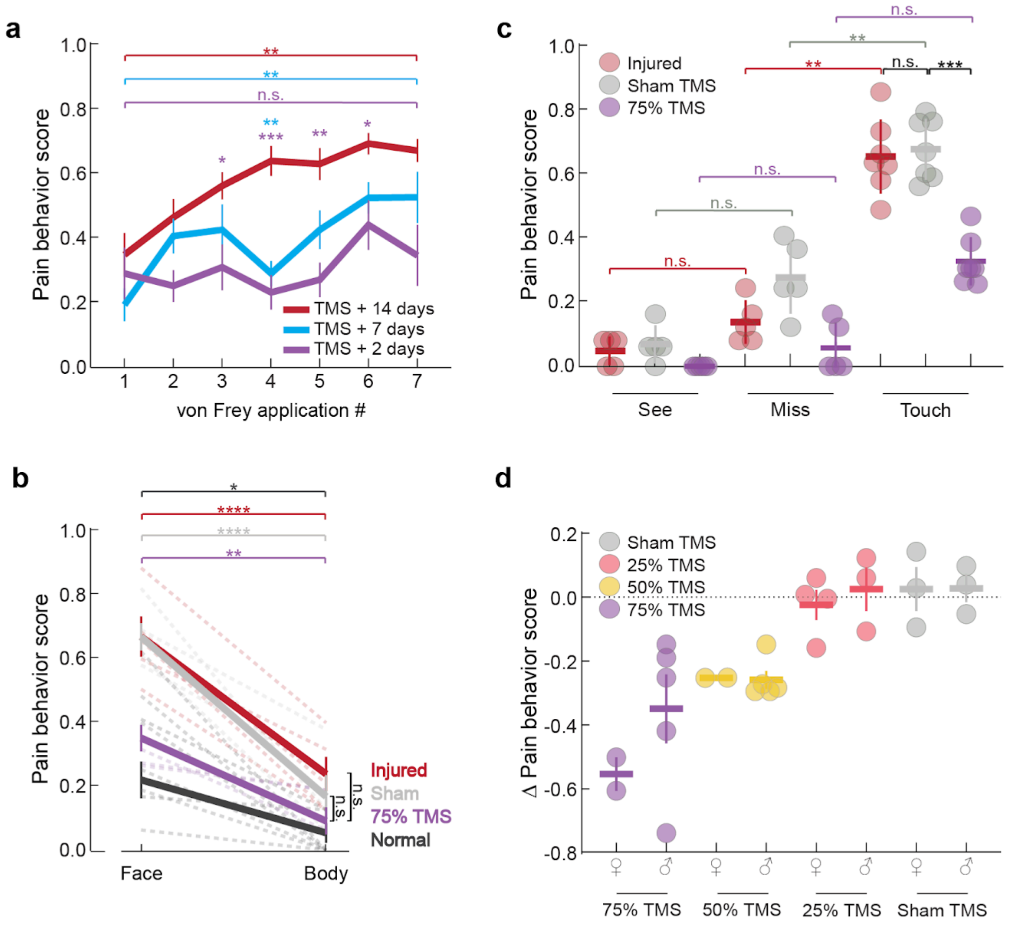

Supplemental Figure 4 | Pain scores were comparable across female and male mice after TMS treatment and were driven by the touches to the face with the von Frey filament.

(a) Within individual testing sessions, pain sensitivity rose in response to multiple applications of a von Frey filament at 7 and 14 days but not 2 days after TMS treatment. Mean ± s.e.m. pain behavior scores in response to successive touches to the face with a von Frey filament during a single testing session, evaluated at 2 d (red), 7d (blue), or 14 d (purple) after TMS treatment at 50% or 75% of motor threshold (Repeated measures two-way ANOVA: von Frey applications #, $p<0.0001$; days after treatment, $p<0.0001$; application # × days post treatment interaction, $p=0.084$; n=15 mice).

bioRxiv preprint doi: https://doi.org/10.64898/2026.07.01.735554; this version posted July 7, 2026. The copyright holder for this preprint (which was not certified by peer review) is the author/funder, who has granted bioRxiv a license to display the preprint in perpetuity. It is made available under a CC-BY 4.0 International license.

(b) TMS treatment led to reduced nocifensive behaviors in response to touches with a von Frey filament to the face but not to the body of the mouse. Plotted are mean ± s.e.m. pain behavior scores, evaluated after a touch to the injured face or body, at ~7 days before injury (*Normal*), 21 days after injury (*Injured*), or 2 days after real (*75% TMS*) or sham (*Sham*) TMS treatment (n=5 mice per condition. Colors denote different treatment conditions. Black: Normal. Red: Injured. Gray: Sham TMS. Purple: 75% motor threshold TMS). (Repeated Measures two-way ANOVA; Condition or treatment (Normal, Injured, Sham TMS, 75% TMS): $p<0.0001$; Face *vs.* body: $p<0.0001$; Condition × face/body interaction: $p=0.009$; *$p<0.05$, **$p<0.01$, ****$p<0.0001$, Fisher's least significant difference (LSD) test with a Holm-Bonferroni correction for multiple comparisons).

(c) Nociceptive stimuli are the primary drivers of mouse nocifensive responses, rather than the sight of nearby or approaching von Frey filaments. Plotted are mean ± s.d. behavior scores of injured mice before (*Injured*) or after sham (*Sham TMS*) or real TMS treatment at 75% of motor threshold (*75% TMS*), evaluated at the time at which the von Frey filament first appeared in the mouse's field of view (*See*), in cases when the experimenter missed the target region (*Miss*), or in response to a successful touch of the von Frey filament (*Touch*) (n=5-7 mice per group; repeated measures two-way ANOVA; $p<0.0001$; Fisher's least significant difference (LSD) post hoc test with Holm-Bonferroni correction; **$p<0.01$, ***$p<0.001$ after adjustment for multiple comparisons).

(d) Male and female mice had comparable treatment responses to TMS. Plotted are mean ± s.e.m. changes in the pain behavior scores of individual mice, evaluated over the period extending from ~7 days before TMS treatment to 2 days afterward, as scored following a touch to the face with the von Frey filament. Data points show results from individual mice, following TMS treatment at the following percentages of motor threshold: *75% TMS* (purple, n=2 female and 5 male mice); *50% TMS* (yellow, n=2 female and 5 male mice); *25% TMS* (orange, n=4 female and 3 male mice); *Sham TMS*

bioRxiv preprint doi: https://doi.org/10.64898/2026.07.01.735554; this version posted July 7, 2026. The copyright holder for this preprint (which was not certified by peer review) is the author/funder, who has granted bioRxiv a license to display the preprint in perpetuity. It is made available under a CC-BY 4.0 International license.

(light gray, n=3 female and 3 male mice). (Repeated measures two-way ANOVA; sex: p=0.27; TMS dose: p=0.001; sex × TMS dose interaction: p=0.46).

bioRxiv preprint doi: https://doi.org/10.64898/2026.07.01.735554; this version posted July 7, 2026. The copyright holder for this preprint (which was not certified by peer review) is the author/funder, who has granted bioRxiv a license to display the preprint in perpetuity. It is made available under a CC-BY 4.0 International license.

Supplemental Figure 5

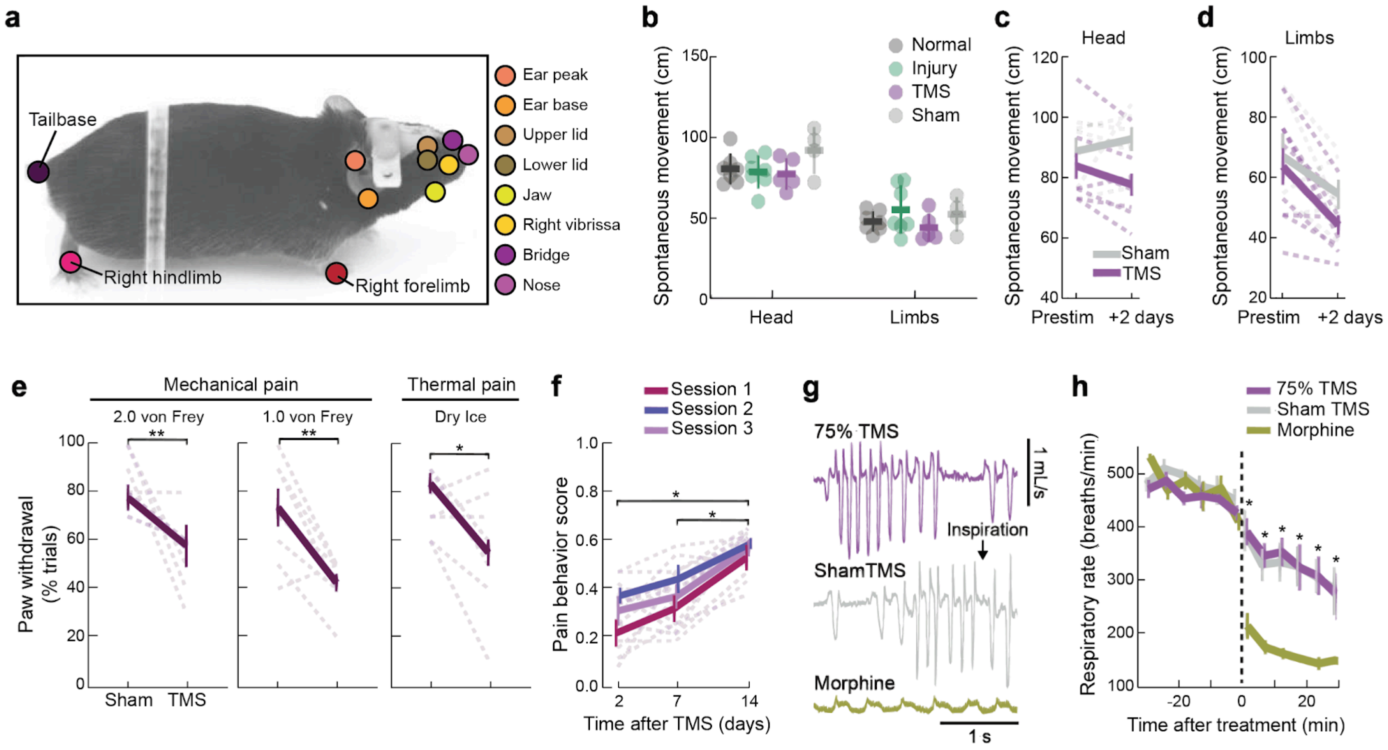

Supplemental Figure 5 | TMS reduced pain-associated behaviors without impairing spontaneous movement or respiratory rates.

(a) Example video frame from a freely moving mouse, overlaid with DeepLabCut markers used to track normal or pain-related head movements (*e.g.,* reflexive withdrawal or grooming), as well as limb movements during locomotion. We tracked keypoints on all four limbs, the base and tip of the tail, and 11 points on the head (nose, jaw, bridge of the nose, left and right vibrissa pads, right and left ear peaks, and the top and bottom points of both the right and left eyelids). We interpolated these positional measurements when body parts were transiently out of view of the camera as the mouse explored its environment. Before each sensory testing session (**Fig. 1e**), we recorded the mouse's behavior for 3 min. To quantify the total amount of head movement during this period, we computed the total mean distance traveled by the keypoints on the nose, nose bridge, vibrissa pads, ear peaks, and jaw. We estimated the mouse's net locomotor distance traveled based on its limb displacements.

bioRxiv preprint doi: https://doi.org/10.64898/2026.07.01.735554; this version posted July 7, 2026. The copyright holder for this preprint (which was not certified by peer review) is the author/funder, who has granted bioRxiv a license to display the preprint in perpetuity. It is made available under a CC-BY 4.0 International license.

(b) Levels of spontaneous head and limb movements were unaffected by TMS treatment at 75% of motor threshold. Plotted are mean ± s.d. distances traveled by the heads and limbs of the mice (n=4–7 mice per group), with each data point showing the mean result for a single mouse, averaged across 2 consecutive testing sessions. (*Normal*: average results for each mouse tested twice at least 7 days after headbar implantation but no more than two days prior to the nerve injury; *Injury*: average results for each mouse at 14 and 21 days after nerve injury; *real and sham TMS groups*: average results for each mouse at 2 and 7 days after treatment; ANOVA across conditions for head movements; p=0.23; ANOVA across conditions for limb movements; p=0.57).

(c, d) TMS produced a subtle reduction in spontaneous head movements but not locomotor distance, suggesting TMS might selectively reduce spontaneous pain in addition to evoked pain. TMS-treated mice exhibited reduced spontaneous head movements, **(c)**, at 2 days post-treatment relative to pre-treatment baseline levels. Spontaneous locomotion, **(d)**, decreased similarly after treatment in mice that received real or sham TMS. Plotted are mean ± s.e.m. distances traveled; thin lines connect results for individual mice from pre-treatment to 2 days post-treatment. **(c)**, Repeated measures two-way ANOVA for head movements, Prestim *vs.* 2 days post-treatment: p=0.92; TMS *vs.* Sham: p=0.02; Interaction: p=0.31; Fisher's LSD post hoc test with Holm-Bonferroni correction for multiple comparisons, *p<0.05. **(d)**, Repeated measures two-way ANOVA for limb movements, Prestim *vs.* 2 days post-treatment: p=0.001; TMS *vs.* Sham: p=0.33; Interaction: p=0.41). (n=4–7 mice per group).

(e) TMS reduced acute pain sensitivity. We applied mechanical and cold nociceptive stimuli to an uninjured hindlimb 30 min after real or sham TMS treatment at 75% of motor threshold. Plotted are the mean ± s.e.m. (n=8 mice) percentage of trials in which mice withdrew from the painful stimulus (n=10 Trials per stimulus). Dashed lines: results from individual mice. (Wilcoxon signed-rank test for each stimulus; 2.0 g von Frey filament: p=0.008; 1.0 g filament: p=0.008; dry ice: p=0.04).

bioRxiv preprint doi: https://doi.org/10.64898/2026.07.01.735554; this version posted July 7, 2026. The copyright holder for this preprint (which was not certified by peer review) is the author/funder, who has granted bioRxiv a license to display the preprint in perpetuity. It is made available under a CC-BY 4.0 International license.

(f) Mice did not desensitize to TMS over repeated administrations. Plotted are mean ± s.e.m. pain scores at days 2, 7, and 14 following each of three sessions of TMS treatment applied at 75% of motor threshold. Successive TMS sessions were 4 weeks apart. Dashed lines: results for each of n=5 individual mice. Repeated measures two-way ANOVA showed that pain scores varied with the time elapsed since TMS treatment ($p<0.0001$) but were statistically indistinguishable across the 3 sessions ($p=0.2$). (*$p<0.05$; Fisher's LSD post hoc test with Holm-Bonferroni correction for multiple comparisons).

(g, h) TMS, despite requiring MOR signaling for analgesia induction, did not cause respiratory depression. As deaths from opioid overdose result mainly from respiratory depression, we used whole-body plethysmography to check for possible respiratory side effects of TMS. Example airflow traces, (**g**), and mean ± s.d. baseline and post-treatment respiratory rates, (**h**), are shown for mice that received either sham or real (75% of motor threshold) TMS or 20 mg/kg morphine (a MOR agonist). The morphine group served as a positive control and received a dosage known to cause respiratory depression in mice. Vertical dashed line: time of treatment delivery. Repeated measures two-way ANOVA (n=6 mice per group; time: $p<0.0001$, treatment: $p<0.001$; Time × treatment interaction: $p<0.0001$) revealed significant effects of morphine compared to either TMS protocol. (*$p<0.05$ denotes time points at which the morphine group differed from the TMS groups using Fisher's LSD post hoc test with a Holm-Bonferroni correction for multiple comparisons).

bioRxiv preprint doi: https://doi.org/10.64898/2026.07.01.735554; this version posted July 7, 2026. The copyright holder for this preprint (which was not certified by peer review) is the author/funder, who has granted bioRxiv a license to display the preprint in perpetuity. It is made available under a CC-BY 4.0 International license.

Supplemental Figure 6

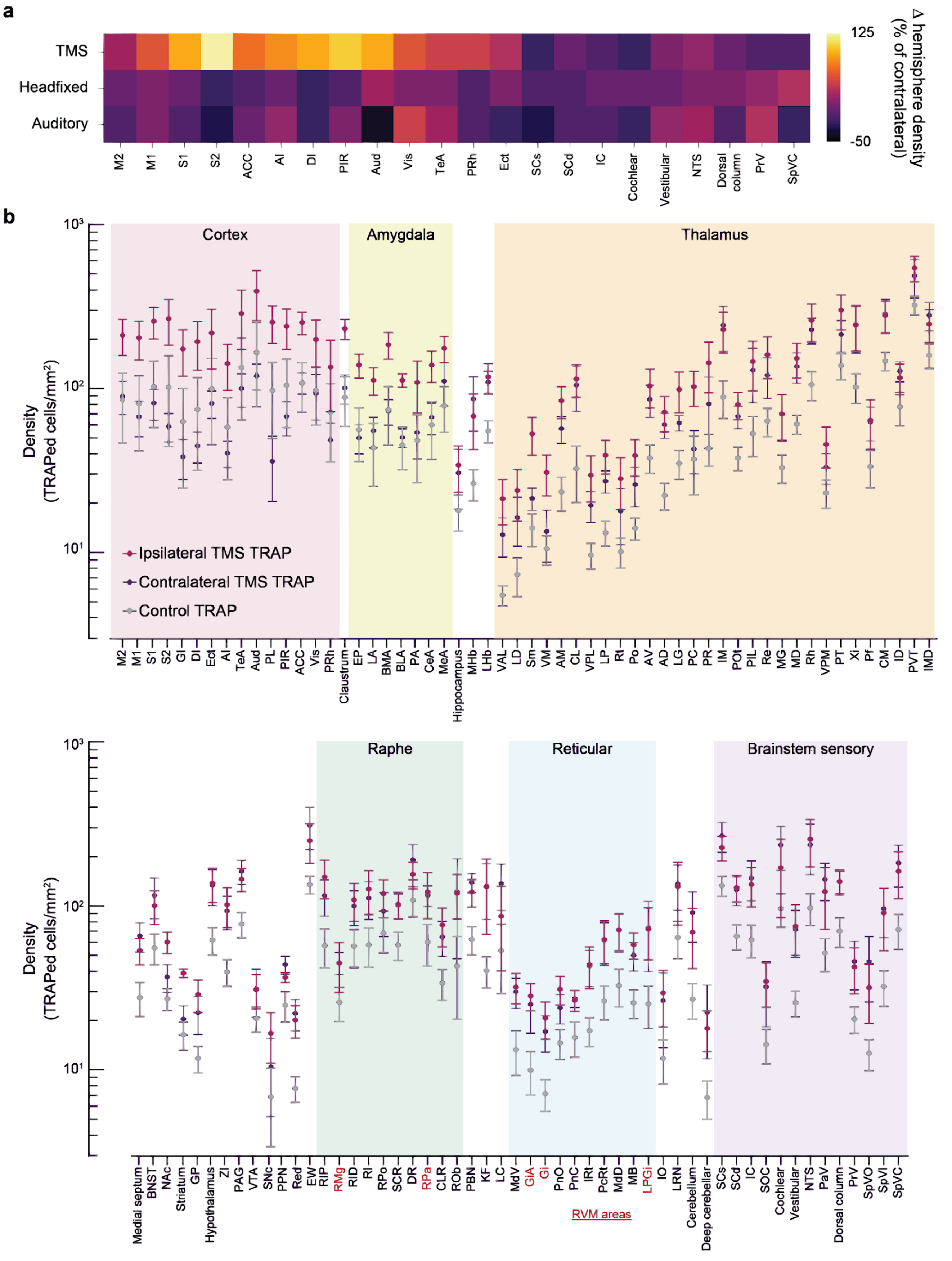

bioRxiv preprint doi: https://doi.org/10.64898/2026.07.01.735554; this version posted July 7, 2026. The copyright holder for this preprint (which was not certified by peer review) is the author/funder, who has granted bioRxiv a license to display the preprint in perpetuity. It is made available under a CC-BY 4.0 International license.

Supplemental Figure 6 | Genetic trapping studies show motor cortical TMS activates neurons ipsilaterally in the neocortex and amygdala and bilaterally in the brainstem.

(a) Motor cortical TMS activates neurons in regions of the ipsilateral neocortex (*TMS*), whereas mere placement of the mouse into the TMS apparatus (*Headfixed*) or exposure of the animal to the sounds produced by the TMS pulses (*Auditory*) do not. The color plot shows, for individual brain areas in each of these three groups of mice, the percentage difference in the mean density of trapped neurons in the hemisphere ipsilateral to the site of TMS, relative to the mean value in the contralateral hemisphere. For the two control groups of mice that received no genuine TMS pulses, we assigned the right hemisphere to the ipsilateral group and the left hemisphere to the contralateral group, just as for the experimental group of mice. (Repeated measures two-way ANOVA; Group (TMS *vs.* Headfixed *vs.* Auditory): p=0.001; Brain areas: p=0.15; Group × Brain area Interaction: p=0.4; n=3 mice per group).

(b) Expanded set of results supporting **Fig. 2c**, showing that neurons were trapped ipsilaterally to the site of stimulation in the neocortex and amygdala and bilaterally in thalamic and brainstem regions. Plotted are mean ± s.e.m. densities of genetically trapped neurons across 109 brain regions in n=5 ipsilateral and contralateral brain hemispheres of mice that received TMS (75% of motor threshold) and in n=10 hemispheres from control mice that were headfixed but did not receive TMS.

Abbreviations: AD: Anterodorsal thalamus, AI: Agranular insular cortex, AM: Anteromedial thalamus, ACC: Anterior cingulate cortex, Aud: Auditory cortex, AV: Anteroventral thalamus, BLA: Basolateral amygdala, BMA: Basomedial amygdala, BNST: Bed nucleus of the stria terminalis, CeA: Central amygdala, CL: Centrolateral thalamus, CLR: Central linear raphe, CM: Centromedian thalamus, DI: Dysgranular insular cortex, DR: Dorsal raphe, Ect: Ectorhinal cortex, EP: Endopiriform, EW: Edinger-Westphal, GI: Granular insular cortex, Gi: Gigantocellular reticular formation, GiA: Gigantocellular reticular formation, alpha part, GP: Globus pallidus, IC: Inferior colliculus, ID: Interanterodorsal thalamus, IM: Interanteromedial thalamus, IMD: Intermediodorsal thalamus, IO:

bioRxiv preprint doi: https://doi.org/10.64898/2026.07.01.735554; this version posted July 7, 2026. The copyright holder for this preprint (which was not certified by peer review) is the author/funder, who has granted bioRxiv a license to display the preprint in perpetuity. It is made available under a CC-BY 4.0 International license.

Inferior olive, IRt: Intermediate reticular formation, KF: Kölliker-Fuse, LA: Lateral amygdala, LC: Locus coeruleus, LD: Lateral dorsal thalamus, LHb: Lateral habenula, LG: Lateral geniculate thalamus, LP: Lateral posterior thalamus, LRN: Lateral reticular formation, LPGi: Lateral paragigantocellular reticular formation, M1: Primary motor cortex, M2: Secondary motor cortex, MB: Midbrain reticular formation, MD: Medial dorsal thalamus, MeA: Medial amygdala, MG: Medial geniculate thalamus, MHb: Medial habenula, MdD: Medullary reticular formation, dorsal, MdV: Medullary reticular formation, ventral, NAc: Nucleus accumbens, NTS: Nucleus of the solitary tract, PAG: Periaqueductal gray, PA: Posterior amygdala, PaV: Paratrigeminal, PC: Paracentral thalamus, PBN: Parabrachial, PcRt: Parvicellular reticular formation, Pf: Parafascicular thalamus, PIL: Posterior intralaminar thalamus, PIR: Piriform cortex, PL: Prelimbic cortex, PnC: Pontine reticular formation, caudal, PnO: Pontine reticular formation, oral, Po: Posterior thalamus, POt: Posterior triangular thalamus, PPN: Pedunculopontine, PRh: Perirhinal cortex, PT: Paratenial thalamus, PVT: Paraventricular thalamus, Re: Reuniens nucleus, RID: Interpeduncular raphe, RI: Interfascicular raphe, RIP: Interpositus raphe, RMg: Raphe magnus, ROb: Raphe obscurus, RPa: Raphe pallidus, RPo: Raphe pontis, Rh: Rhomboid thalamus, Rt: Reticular thalamus, S1: Primary somatosensory cortex, S2: Secondary somatosensory cortex, SCd: Superior colliculus, dorsal, SCs: Superior colliculus, superficial, SCR: Superior central raphe, Sm: Submedial thalamus, SNc: Substantia nigra compacta, SOC: Superior olivary complex, SpVC: Spinal trigeminal nucleus caudalis, SpVI: Spinal trigeminal nucleus interpolaris, SpVO: Spinal trigeminal nucleus oralis, TeA: Temporal association cortex, VAL: Ventral anterior-lateral thalamus, Vis: Visual cortex, VM: Ventral medial thalamus, VPL: Ventral posterolateral thalamus, VPM: Ventral posteromedial thalamus, VTA: Ventral tegmental area, Xi: Xiphoid thalamus, ZI: Zona incerta

bioRxiv preprint doi: https://doi.org/10.64898/2026.07.01.735554; this version posted July 7, 2026. The copyright holder for this preprint (which was not certified by peer review) is the author/funder, who has granted bioRxiv a license to display the preprint in perpetuity. It is made available under a CC-BY 4.0 International license.

Supplemental Figure 7

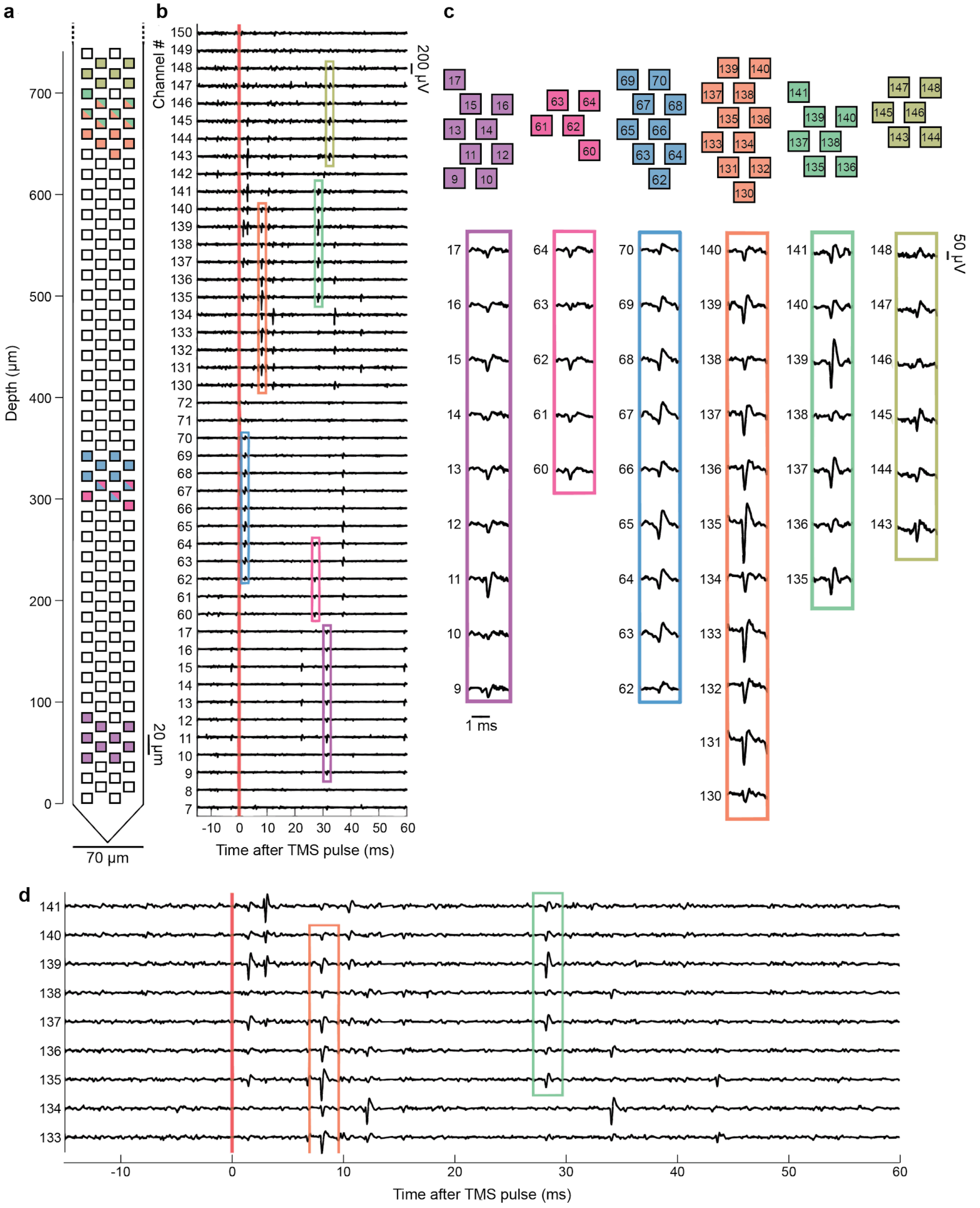

bioRxiv preprint doi: https://doi.org/10.64898/2026.07.01.735554; this version posted July 7, 2026. The copyright holder for this preprint (which was not certified by peer review) is the author/funder, who has granted bioRxiv a license to display the preprint in perpetuity. It is made available under a CC-BY 4.0 International license.

Supplemental Figure 7 | Spike waveforms of RVM neurons recorded with Neuropixels probes during TMS pulses.

(a, b) Electrical activity observed on multiple Neuropixels recording channels after a single pulse of TMS (red vertical line). (**a**) Schematic of a Neuropixel probe. Recording sites on which the electrical dynamics of 6 different example RVM neurons (from the light blue-labeled probe in **Fig. 4b**) were observed are marked in 6 corresponding colors. (**b**) Band-pass filtered (300 Hz–10 kHz) electrical traces from 150 different recording channels reveal the responses of individual RVM neurons to a single TMS pulse. Auditory responses to the clicking sound of a TMS pulse appeared right after stimulation (2–7 ms after the pulse, as illustrated by the blue-labeled cell, likely relayed monosynaptically from the cochlear nuclei[148]), followed by activity driven by Layer 5 pyramidal tract neurons that project monosynaptically to the RVM (5–15 ms after the pulse, within the range of delays reported in past studies[149,150], as illustrated by the orange-labeled cell). Subsequent waves of incoming excitation were likely polysynaptic and probably included a mix of auditory and TMS-driven signals (15 ms to >50 ms after the pulse, as illustrated by the pink-, purple-, green-, and olive-labeled cells).

(c) Example traces of spike waveforms (*lower*) recorded across multiple channels of the Neuropixels probe (*upper*) for the same 6 color-corresponding neurons shown in **a** and **b**. The color associated with each set of spike waveforms conveys the location of the corresponding recording sites, as shown in **a**.

(d) Illustrative subset of electrical traces from recording channels 133 to 141, shown on an expanded scale. Even though spike waveforms from different cells can be detected on overlapping sets of recording sites, spikes from the different neurons can still be differentiated based on their distinct footprints across the Neuropixels probe, as illustrated in panel **c**.
